# Identification of key candidate genes for IgA nephropathy using machine learning and statistics based bioinformatics models

**DOI:** 10.1038/s41598-022-18273-x

**Published:** 2022-08-17

**Authors:** Md. Al Mehedi Hasan, Md. Maniruzzaman, Jungpil Shin

**Affiliations:** 1 School of Computer Science and Engineering, The University of Aizu, Aizuwakamatsu, Fukushima, 965-8580 Japan; 2 Statistics Discipline, Khulna University, Khulna, 9208 Bangladesh

**Keywords:** Computational biology and bioinformatics, Machine learning, Microarrays, Proteome informatics, Statistical methods

## Abstract

Immunoglobulin-A-nephropathy (IgAN) is a kidney disease caused by the accumulation of IgAN deposits in the kidneys, which causes inflammation and damage to the kidney tissues. Various bioinformatics analysis-based approaches are widely used to predict novel candidate genes and pathways associated with IgAN. However, there is still some scope to clearly explore the molecular mechanisms and causes of IgAN development and progression. Therefore, the present study aimed to identify key candidate genes for IgAN using machine learning (ML) and statistics-based bioinformatics models. First, differentially expressed genes (DEGs) were identified using limma, and then enrichment analysis was performed on DEGs using DAVID. Protein-protein interaction (PPI) was constructed using STRING and Cytoscape was used to determine hub genes based on connectivity and hub modules based on MCODE scores and their associated genes from DEGs. Furthermore, ML-based algorithms, namely support vector machine (SVM), least absolute shrinkage and selection operator (LASSO), and partial least square discriminant analysis (PLS-DA) were applied to identify the discriminative genes of IgAN from DEGs. Finally, the key candidate genes (FOS, JUN, EGR1, FOSB, and DUSP1) were identified as overlapping genes among the selected hub genes, hub module genes, and discriminative genes from SVM, LASSO, and PLS-DA, respectively which can be used for the diagnosis and treatment of IgAN.

## Introduction

Immunoglobulin-A-nephropathy (IgAN) is one of the major public health problems. IgAN is also known as Berger’s disease^1,2^. It is a kidney disease caused by the accumulation of IgAN deposits in the kidneys, which causes inflammation and damage to the kidney tissues. IgAN is the most common primary glomerulonephritis that can progress to renal failure worldwide^3,4^. IgAN is sometimes associated with different kinds of diseases such as heart disease^5,6^, liver cirrhosis^6,7^, coeliac disease^6,8^, skin disease^6^. About, 20–47% of primary glomerular diesases are responsible for IgAN, which is mainly characterized by hypertension, hematuria, proteinuria, and failure of the renal^9,10^. About 20–40% of people with IgAN have end-stage renal disease after 10–20 years^11^. The overall prevalence of IgA nephropathy varies from regions to regions^12^. The highest prevalence’s of IgAN are found in Asia region (especially, in China and Japan) and its prevalence has been diagrammatically increased over past three decades^13–15^. It is noted that the risks of deaths have been increased among patients with IgAN^16^. As a result, we need to know the molecular mechanism about the development and progression of IgAN in order to diagnose IgAN patients properly and decrease the death rate. However, molecular mechanism can be studied properly by knowing the key genes or biomarkers for the development and progression of IgAN. Despite numerous studies examining the molecular characteristics of IgAN, the mechanism underlying IgAN development and progression remains a challenging issue^15^. Therefore, it is urgent to propose an effective tool for determining potential or key candidate genes of IgAN in order to understand molecular mechanism of IgAN.

Bioinformatics analysis is a powerful approach for predicting molecular pathways and gene connections. This approach is widely used to predict novel candidate genes and pathways associated with different cancers like breast^17^, gastric^18^, cervical^19^, and so on. Recently, this approach has increasingly revealed the molecular pathways underlying kidney disease^20,21^. Various studies were conducted for the identification of key hub genes for IgAN patients. Qian et al. suggested twenty-one hub genes as well as identified five key candidate genes which were strongly correlated with IgAN patients^10^. Zhang et al. investigated ten hub genes of IgAN and proposed four novel biomarkers that may be played an essential roles in the progression of IgAN and could be used as potential biomarkers for IgAN diagnosis and treatment^20^. Chen et al. suggested six biomarkers that were also related to the pathogenesis of IgAN^22^. Chen et al. also suggested plausible new drugs (thapsigargin, ciclopirox, and ikarugamycin) for the treatment of IgAN^22^. All of these previous studies demonstrated key biomarkers of IgAN using bioinformatics analysis^10,20,22–25^ and showed different gene sets as key candidate genes. All researcher identified their potential biomarkers or genes using only hub genes, determined by the degree of connectivity in the PPI netwrok. In recent years, machine learning (ML)-based techniques have gained more popularity to ease one of the important challenges associated with study of genetic data: extraction of meaningful genes^26–28^. Since the set of identified key genes by existing works are different, there is still some scope to identify genes more confidently using ML and statistics-based bioinformatics models.

**Figure 1:**
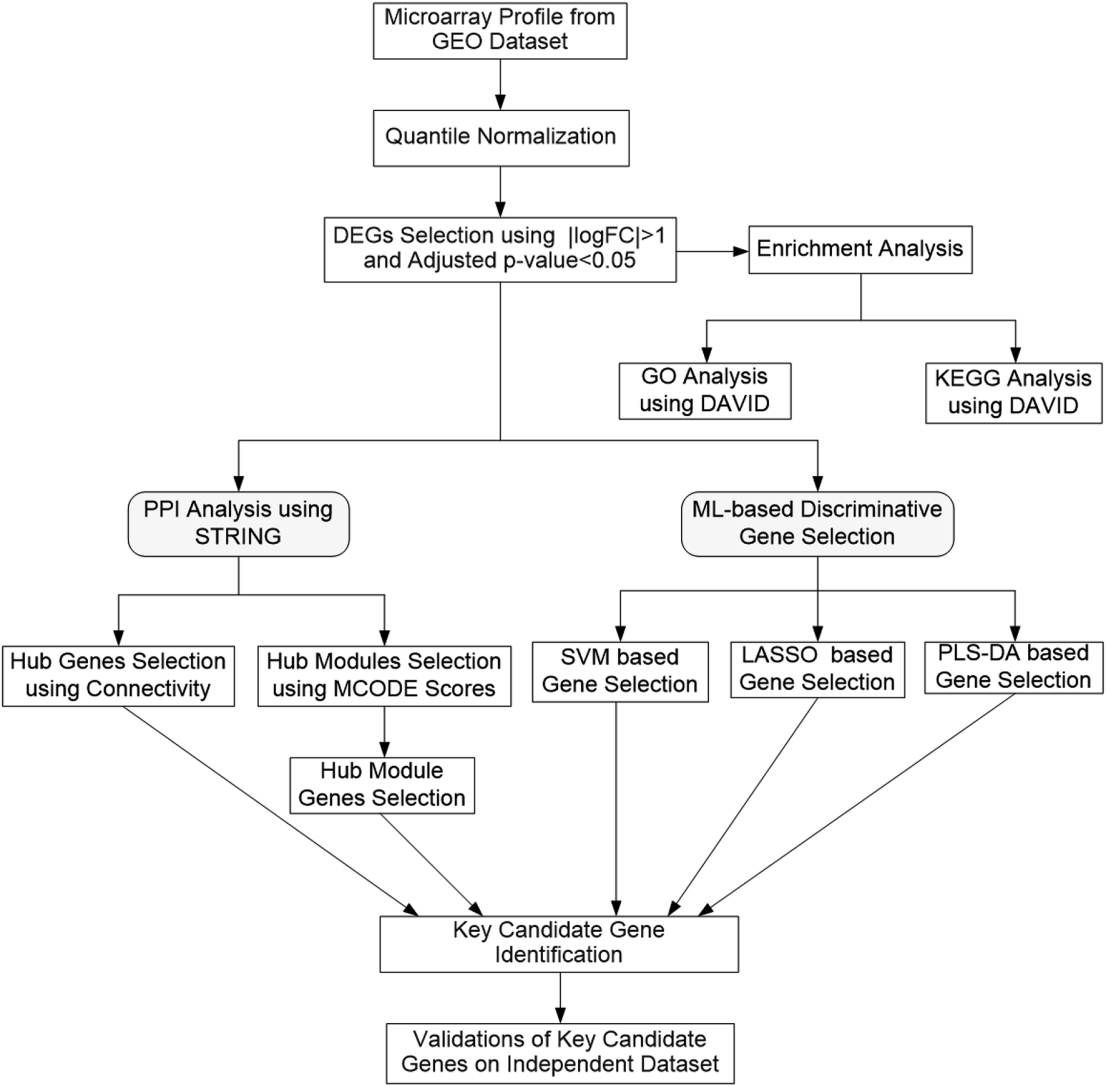
Flowchart of data preparation, processing, analysis, and validation.

In the current study, we selected one microarray gene expression (MGE) dataset from the Gene Expression Omnibus (GEO) database to identify the key candidate genes of IgAN. First, we identified DEGs for IgAN patients. Then, we used Database for Annotation, Visualization, and Integration Discovery (DAVID) to discover the functions of the DEGs and obtained Gene Ontology (GO) and Kyoto Encyclopedia of Genes and Genomes (KEGG) pathway analyses. Using the STRING database, we constructed a protein–protein interaction (PPI) network and identified hub genes from Cytoscape using the degree of connectivity as well as the most potential modules using Molecular Complex Detection (MCODE). We also identified the hub modules and their associated genes from the selected potential modules. We applied three ML-based algorithms to identify the significant genes for IgAN patients. The objective of this research was to determine the potential key candidate genes or biomarkers that can be used to diagnose and treat IgAN. Figure 1 summarized the data preparation, processing, analysis, and validation.

## Results

### Experimental settings

For this experiment, the R-programming language version 4.1.2 was used for all statistical analysis. As the operating system, Windows 10 version 21H1 (build 19043.1151) 64 bit was used. In terms of hardware, an Intel (R) Core (TM) i5-10400 processor with 16 GB of RAM was used. In this study, we used three GEO datasets: GSE93798, GSE116626 and GSE35487. We selected the key candidate genes from the GSE93798 dataset. Another two independent test datasets: GSE116626 and GSE35487 were used for the validation of key candidate genes.

### Identification of DEGs

**Figure 2:**
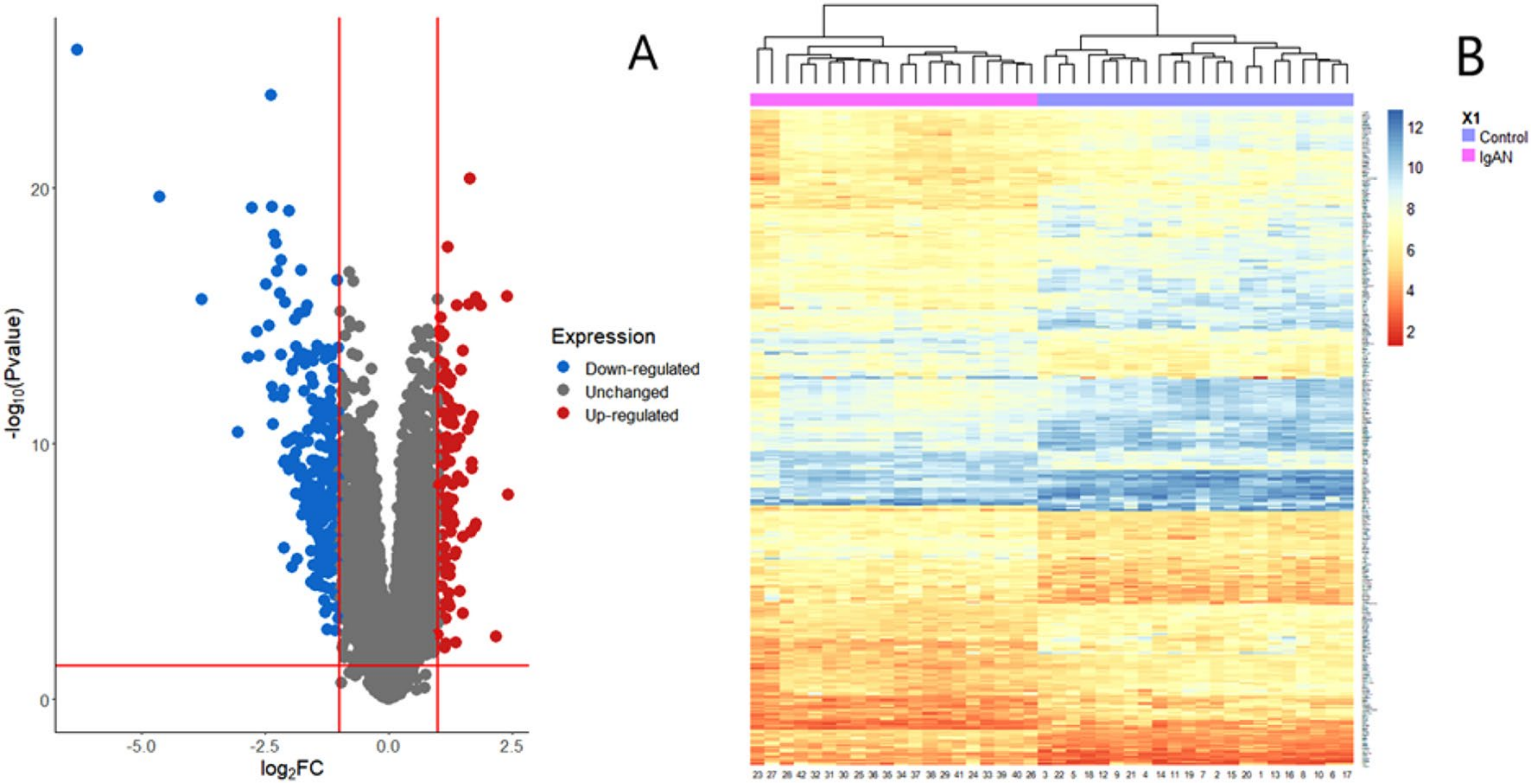
Identification and hierarchical clustering of DEGs for IgAN patients. (**A**) Volcano plot of DEGs which were generated using “ggplot2 version 3.3.6” package in R^63^ (https://cran.r-project.org/package=ggplot2) . Dodger blue represents down-regulated, gray represents no significant genes, and fire brick represents up-regulated DEGs. (**B**) Heatmap of the DEGs for IgAN patients which were generated using “NMF” version 0.24.0 package in R^64^ (https://cran.r-project.org/package=NMF). The horizontal axis shows the number of patients and the vertical axis shows DEGs.

Using the cutoff of adjusted *p*-value $< 0.05$ and $|\text {logFC}|>1$, a total of 348 DEGs were identified for IgAN patients. Among them, 107 genes were up-regulated and 241 genes were down-regulated. The volcano plot and heatmap of the DEGs for IgAN patients and healthy controls was presented in Fig. 2A,B.

### Go term enrichment and KEGG pathway analysis

We imported the DEGs into the DAVID for the enrichment analysis of GO and KEGG pathways. To determine the significant GO terms and KEGG pathway, we considered the cutoff point of *p*-value $< 0.05$. In Table 1, the top five significant GO terms of DEGs for biological process (BP), cellular component (CC), and molecular function (MF) were presented. As in BP, the DEGs were significantly enriched in response to inflammatory, response to camp, cytokine-mediated signaling pathway, cellular response to lipopolysaccharide, and neutrophil chemotaxis. In the CC group, the DEGs were mainly enriched in extracellular exosome, region, space, collagen trimer, and blood microparticle. The MF group GO terms, including transcriptional activator activity, RNA polymerase II transcription regulatory region sequence-specific binding, zinc ion binding, transmembrane transporter activity, and gextra cellular matrix structural constituent conferring tensile strength, were significantly enriched by DEGs.

**Table 1:** GO analysis of DEGs in biological process, cellular component, and molecular function.

	GO ID	Description	Count	*p*-value
BP	GO:0006954	Inflammatory response	32	$8.16 \times 10^{-13}$
	GO:0051591	Response to camp	10	$5.67 \times 10^{-8}$
	GO:0019221	Cytokine-mediated signaling pathway	21	$3.78 \times 10^{-7}$
	GO:0071222	Cellular response to lipopolysaccharide	16	$5.44 \times 10^{-7}$
	GO:0030593	Neutrophil chemotaxis	11	$7.67 \times 10^{-7}$
CC	GO:0070062	Extracellular exosome	93	$2.79 \times 10^{-18}$
	GO:0005576	Extracellular region	70	$8.49 \times 10^{-9}$
	GO:0005615	Extracellular space	62	$1.58 \times 10^{-7}$
	GO:0005581	Collagen trimer	12	$3.03 \times 10^{-7}$
	GO:0072562	Blood microparticle	14	$6.18 \times 10^{-7}$
MF	GO:0005201	Extracellular matrix structural constituent	15	$8.57 \times 10^{-8}$
	GO:0001228	Transcriptional activator activity	27	$1.18 \times 10^{-7}$
	GO:0008270	Zinc ion binding	36	$1.39 \times 10^{-6}$
	GO:0022857	Transmembrane transporter activity	13	$1.41 \times 10^{-5}$
	GO:0030020	Gextra cellular matrix structural constituent conferring tensile strength	7	$5.84 \times 10^{-5}$

Top five items were selected based on *p*-value.

*GO* gene ontology, *BP* biological process, *CC* cellular component, *MF* molecular function.

**Table 2:** KEGG pathway analysis of DEGs.

Pathway ID	Description	Count	*p*-value
hsa00260	Glycine, serine and threonine metabolism	10	$4.50 \times 10^{-7}$
hsa04933	Age-rage signaling pathway in diabetic complications	13	$6.63 \times 10^{-6}$
hsa04974	Protein digestion and absorption	12	$4.90 \times 10^{-5}$
hsa04657	IL-17 signaling pathway	10	$5.45 \times 10^{-4}$
hsa04380	Osteoclast differentiation	11	0.0013

Top five items were selected based on *p*-value.

The analysis of the KEGG pathway for DEGs was showed in Table 2. We observed that DEGs were mainly involved in glycine, serine and threonine metabolism, age-rage signaling pathway in diabetic complications, protein digestion and absorption, IL-17 signaling pathway, and osteoclast differentiation.

### PPI network construction and hub gene selection

**Figure 3:**
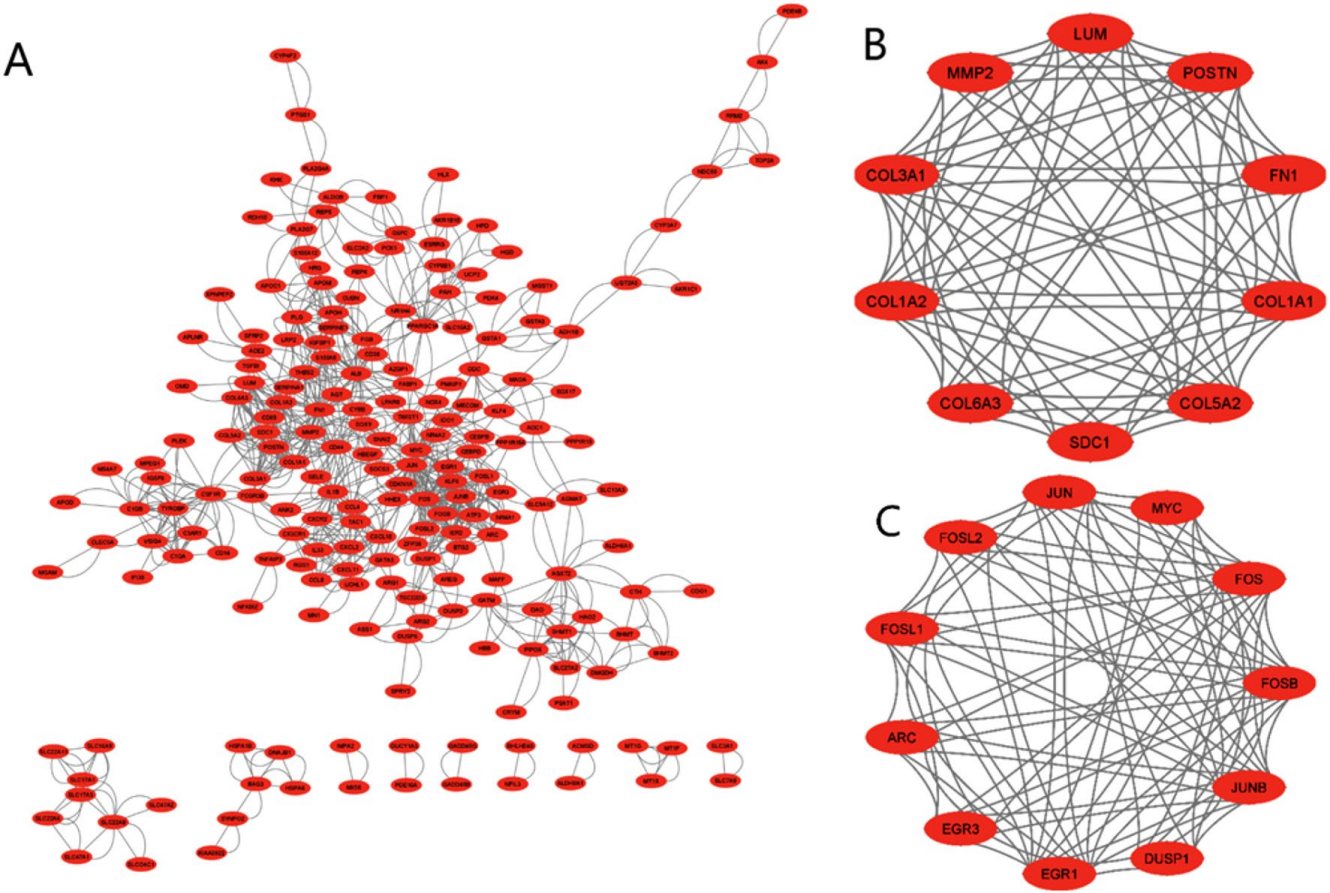
(**A**) PPI network of DEGs, (**B**) Module 1, and (**C**) Module 2. These three figures were generated by Cytoscape 3.9.1^54^ (www.cytoscape.org).

PPI networks TSV data file was obtained from STRING and imported to Cytoscape and built a PPI network with 206 nodes and 880 edges (see Fig. 3A). The hub genes were selected using a degree of connectivity $>18$. Using this cutoff, we selected 19 hub genes which were shown in detail in Table 3.

**Table 3:** List of 19 hub genes which were identified from PPI network based on degree of connectivity.

SN	Gene	Degree	Betweenness	Closeness
1	FOS	50	0.113	0.314
2	JUN	44	0.164	0.326
3	FN1	38	0.113	0.321
4	ALB	34	0.190	0.330
5	IL1B	32	0.234	0.337
6	EGR1	32	0.012	0.280
7	JUNB	30	0.023	0.291
8	CD44	28	0.074	0.310
9	MMP2	28	0.033	0.288
10	MYC	26	0.076	0.315
11	FOSB	26	0.011	0.275
12	COL1A2	24	0.006	0.264
13	TYROBP	22	0.060	0.223
14	CSF1R	22	0.093	0.248
15	COL1A1	22	0.008	0.269
16	CCL4	20	0.062	0.303
17	ATF3	20	0.044	0.264
18	DUSP1	20	0.036	0.262
19	LUM	20	0.013	0.250

### Hub module and its associated gene selection

A total of 13 modules/clusters were built using MCODE with the cutoffs: degree = 2, cluster finding = haircut, nodes score = 0.2, K-score = 2, and max depth = 100. The MCODE scores ranged from 3 to 8.44. We selected two significant modules with cutoffs: MCODE scores $\ge 6$ and number of nodes $\ge 6$ for determining hub module genes (see Table 4). The corresponding PPI network for module 1 and module 2 were showed in Fig. 3B,C. There were 10 genes in module 1 and 11 genes in module 2. We took the union of module 1 and module 2 and got 21 genes which were considered as hub module genes.

**Table 4:** Two modules selected from the PPI network.

Cluster	Score	Nodes	Edges	Node IDs
1	8.44	10	76	COL5A2, POSTN, COL6A3, LUM, COL1A1, SDC1, COL3A1, MMP2, FN1, COL1A2
2	8.40	11	84	DUSP1, JUN, JUNB, EGR3, MYC, FOSL2, FOSB, FOSL1, EGR1, FOS, ARC

Score=density $\times$ no. of nodes.

### Analysis of discriminative gene selection using ML

#### Identifying discriminative genes using SVM

**Figure 4:**
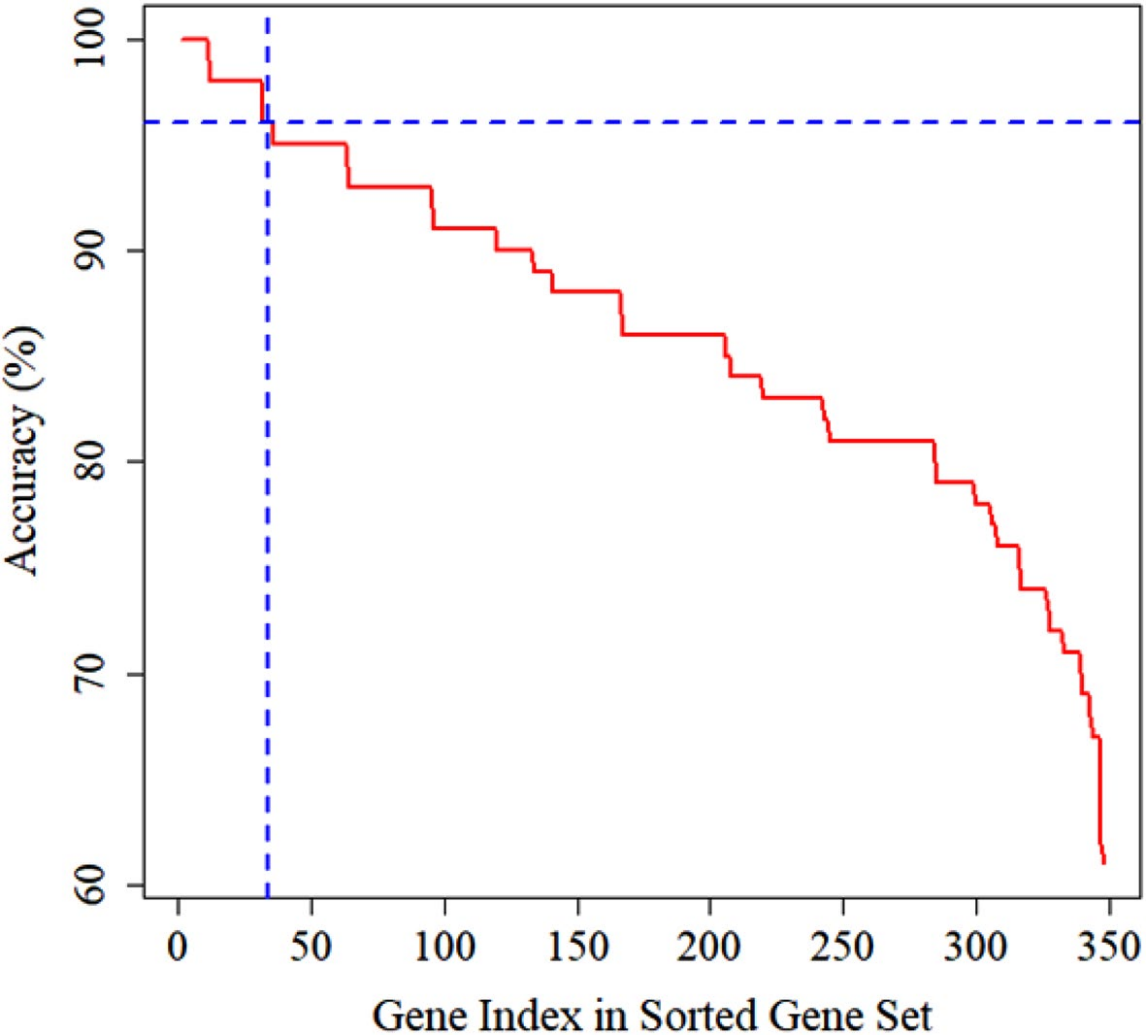
Classification accuracy of SVM for each gene.

We applied SVM with RBF kernel on 348 DEGs and computed the classification accuracy for each gene. The gene selection procedure using SVM was already discussed in “Methods” section. The classification accuracy of each gene had sorted and were showed in Fig. 4. We selected 35 discriminative genes out of 348 DEGs whose classification accuracy was greater than 95.0%.

#### Identifying discriminative genes using LASSO

**Figure 5:**
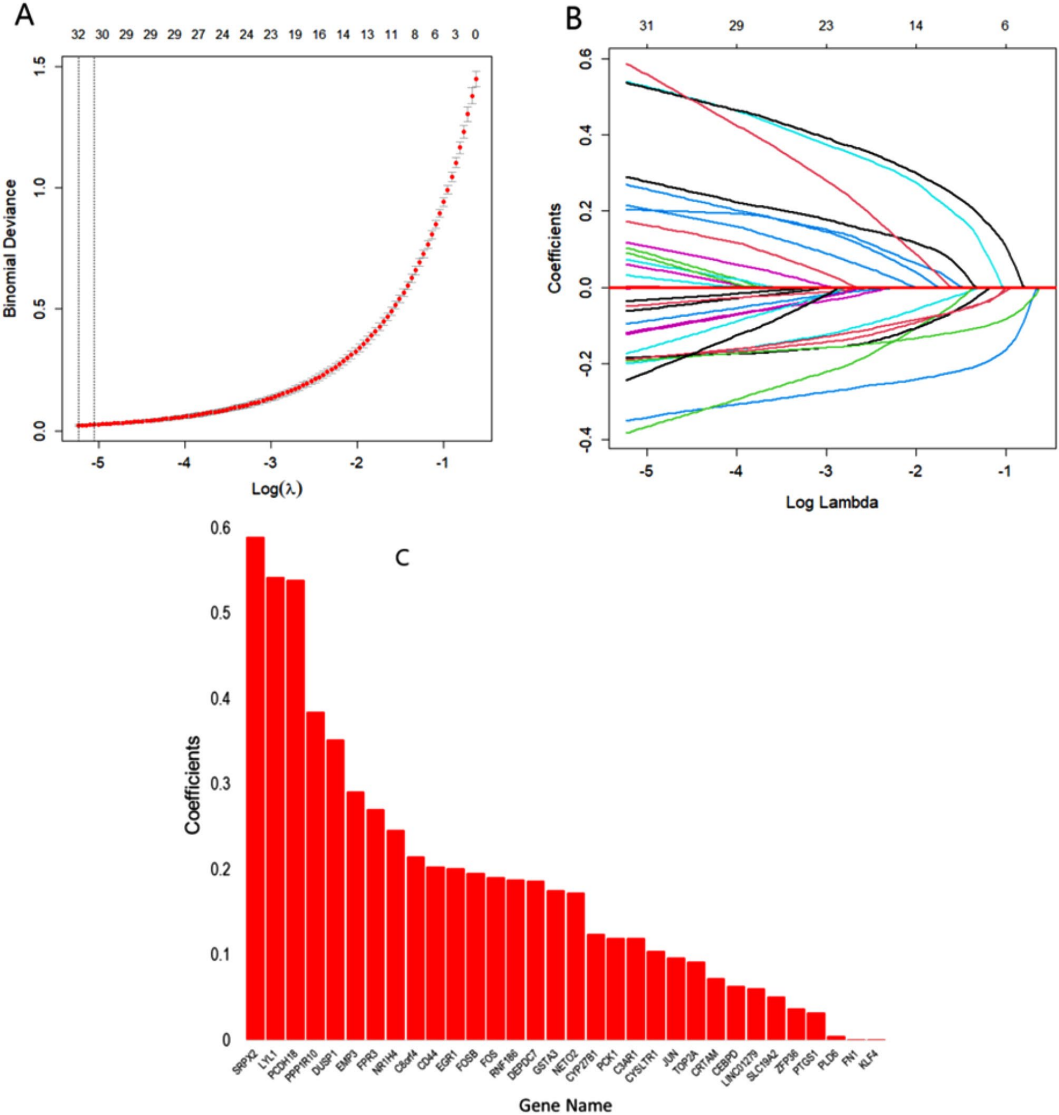
Discriminative gene selected using LASSO-based model by 10 CV: (**A**) A coefficient profile plot was generated against the log (λ) sequence. (**B**) 32 discriminative genes were selected for IgAN. (**C**) Contribution of 32 discriminative genes for IgAN patients.

A total of 348 DEGs were identified between IgAN and control groups to fit LASSO-based logistic regression model. The next step was to determine the optimal values for lambda (λ= 0.008012) using 10-fold CV. Finally, 32 discriminative genes (SRPX2, LYL1, PCDH18, PPP1R10, DUSP1, EMP3, FPR3, NR1H4, C8ORF4, CD44, EGR1, FOSB, FOS, RNF186, DEPDC7, GSTA3, NETO2, CYP27B1, PCK1, C3AR1, CYSLTR1, JUN, TOP2A, CRTAM, CEBPD, LINC01279, SLC19A2, ZFP36, PTGS1, PLD6, FN1, KLF4) with no-zero coefficients were identified in discriminating IgAN and healthy control (see Fig. 5).

#### Identifying discriminative genes using PLS-DA

**Figure 6:**
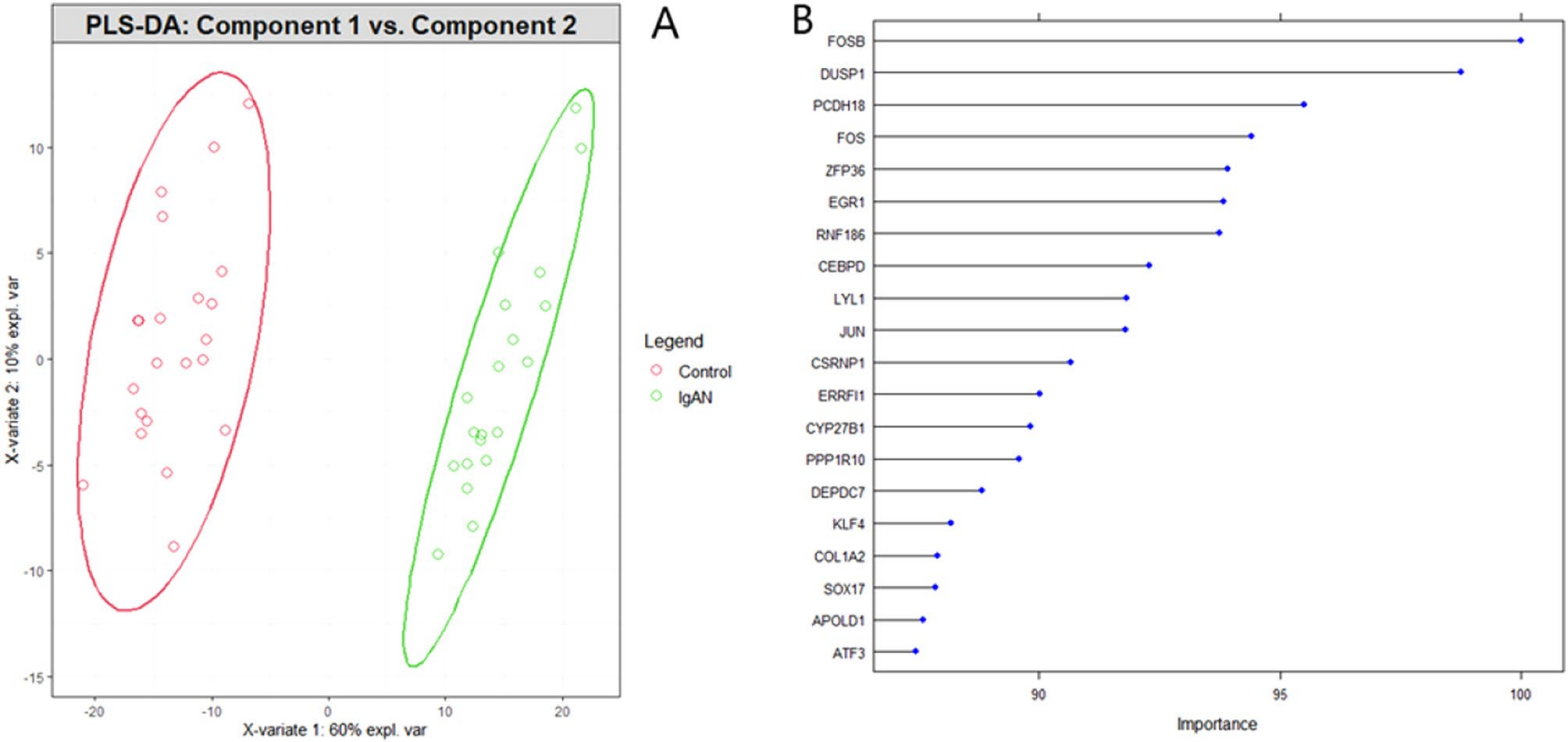
PLS-DA for DEGs: (**A**) Component 1 vs. Component 2. The red points indicate IgAN patients and the green points indicate healthy control; (**B**) Importance of top 20 discriminative genes for IgAN.

PLS-DA was adopted on 348 DEGs to determine the significant genes of IgAN patients. We selected 20 components. Among them, we took the first two PLS-DA components and visualized these two components, which were presented in Fig. 6A. The red points indicated the IgAN patients and the green points indicated the healthy controls (Fig. 6A). PLS-DA can be significantly differentiated IgAN patients from healthy controls. We selected the top 20 most important genes (FOSB, DUSP1, PCDH18, FOS, ZFP36, EGR1, RNF186, CEBPD, LYL1, JUN, CSRNP1, ERRFI1, CYP27B1, PPP1R10, DEPDC7, KLF4, COL1A2, SOX17, APOLD1, and ATF3) for IgAN patients, which were illustrated in Fig. 6B.

### Key candidate genes selection

**Figure 7:**
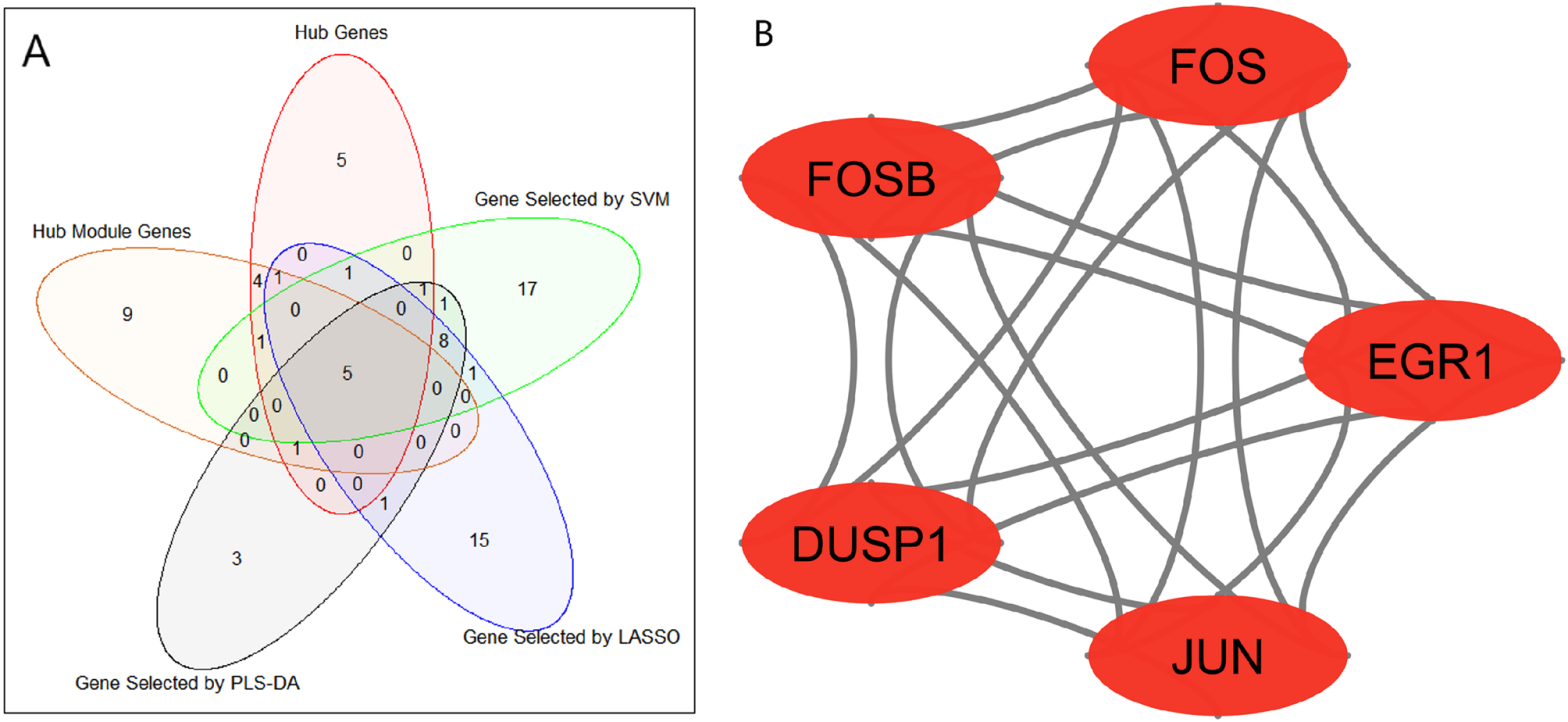
Identification and PPI analysis of key hub genes for IgAN patients. (**A**) Key candidate genes identification from hub module genes, computed from Cytohubba, SVM, LASSO, and PLS-DA. (**B**) PPI analysis of key five candidate genes.

**Figure 8:**
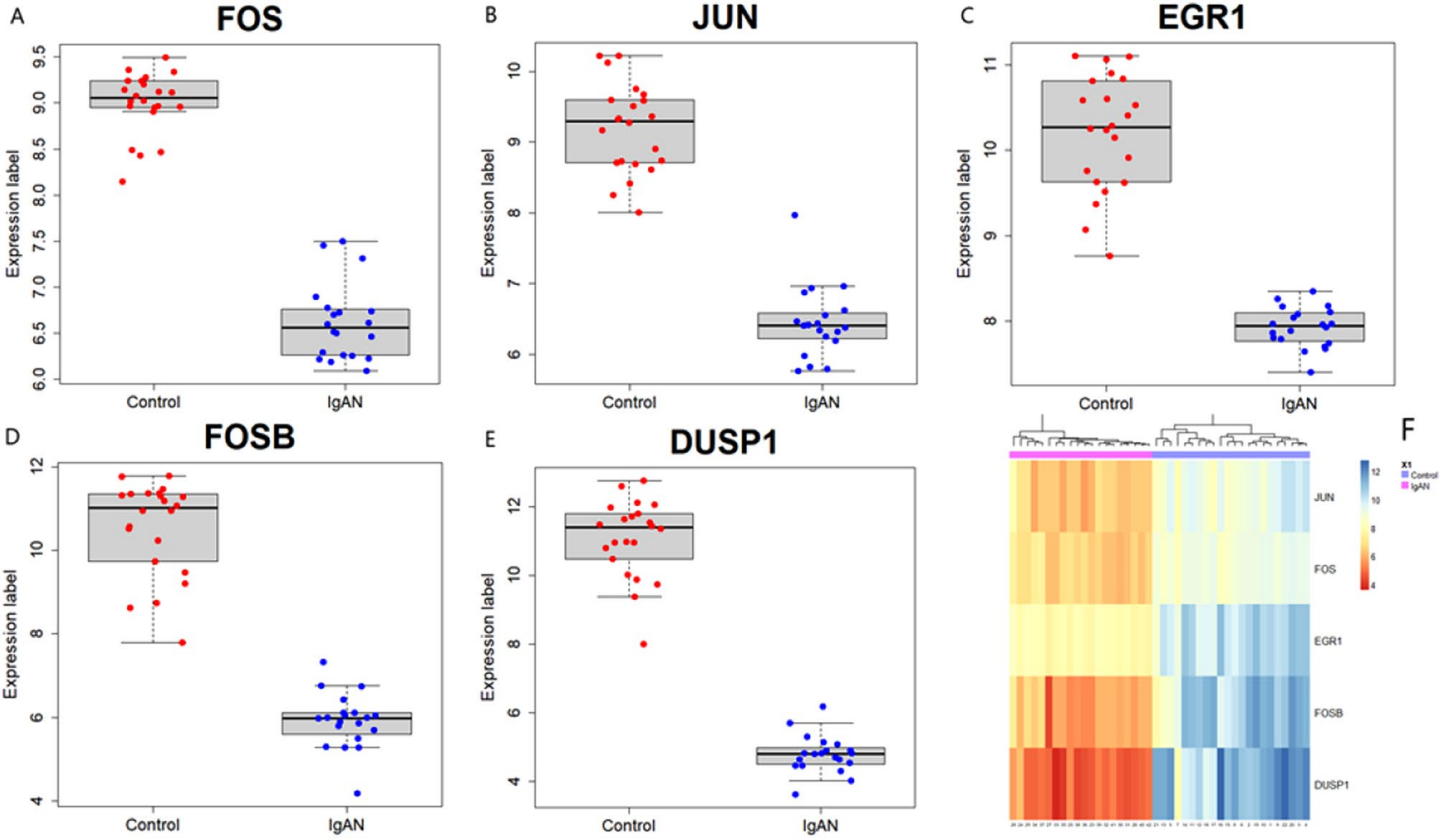
Boxplot of five key candidate genes as (**A**) FOS, (**B**) JUN, (**C**) EGR1, (**D**) FOSB, (**E**) DUSP1 for IgAN patients, and (**F**) Heatmap of the five key candidate genes in renal tissue samples which were generated using “NMF” version 0.24.0 package in R^64^ (https://cran.r-project.org/package=NMF).

The key candidate genes were identified by overlapping genes according to five methods. Among them, three methods were ML-based algorithms (SVM, LASSO, and PLS-DA) for the identification of discriminative genes. The hub genes were identified using the degree of connectivity from the PPI network and hub module genes were from two significant modules. Five key candidate genes (FOS, JUN, EGR1, FOSB, and DUSP1) were selected, which were shown in Fig. 7A, and their PPI network analysis was also shown in Fig. 7B. These five key candidate genes and their probable significance in IgAN indicated that they could be novel therapeutic target genes. We observed that each key candidate gene was significantly differentiated IgAN patients from healthy controls (Fig. 8A–E). We also performed hierarchical clustering for each candidate gene, which was shown in Fig. 8F.

### Validation or confirmation of key candidate genes

The GSE116626 and GSE35487 datasets were used for the validation of key candidate genes. We evaluated five key candidate genes on the basis of area under the curve (AUC), computed from the receiver operating characteristic curve (ROC). For ROC analysis of each gene, the class label (IgAN vs. healthy control) and gene expression labels need to be collected. First, we used leave-one-out cross-validation and employed a logistic regression (LR) model to classify the subjects as either IgAN or healthy controls. After fitting the LR model, we computed AUC values using “pROC” R-package^29^.

**Figure 9:**
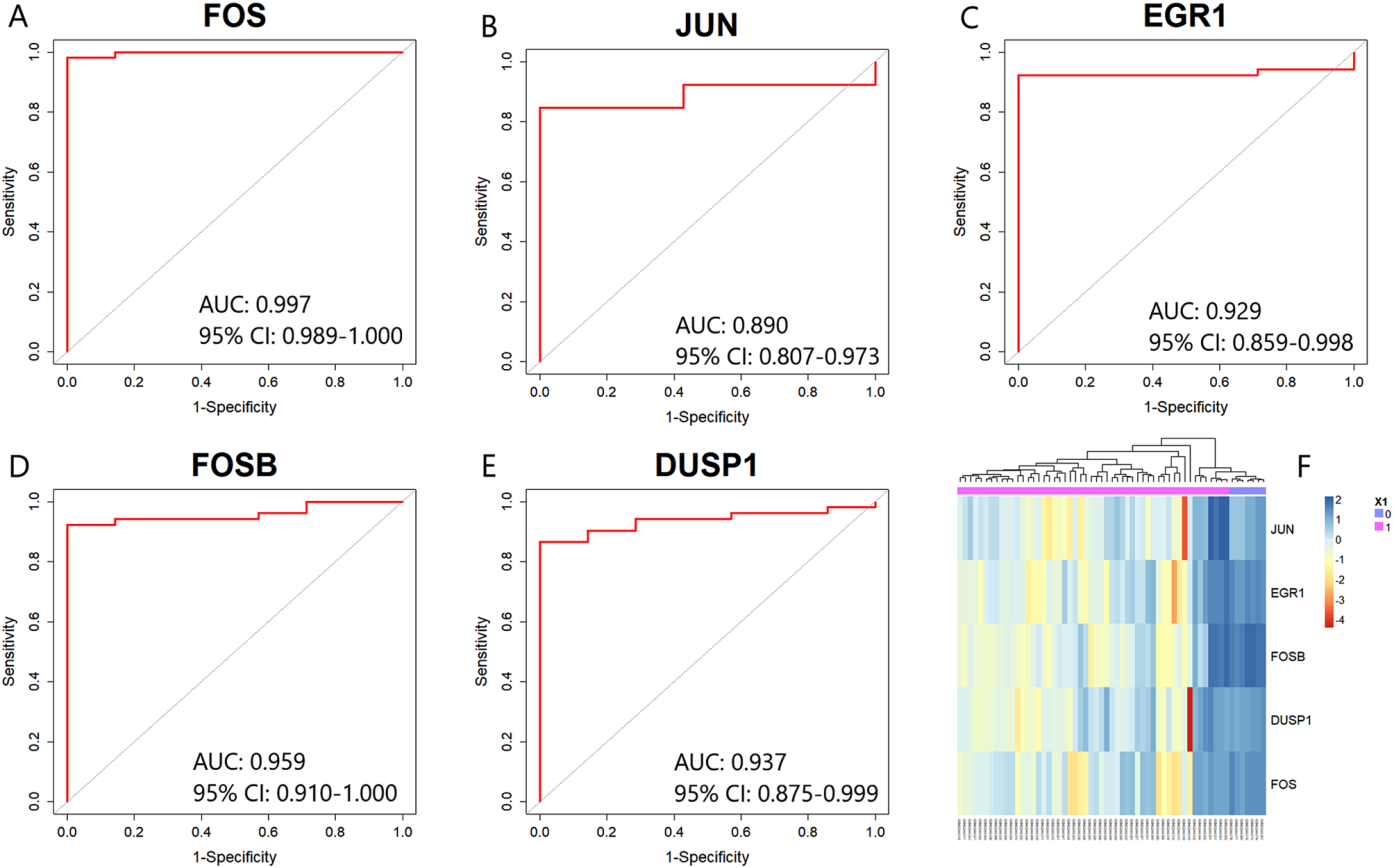
Validation of the five key candidate genes using ROC curves which were generated by pROC package with version 1.18.0 in R^29^ (https://cran.r-project.org/package=pROC) and heatmap for GSE116626 dataset. (**A**) FOS (**B**) JUN (**C**), EGR1 (**D**) FOSB (**E**) DUSP1 (**F**) Heatmap of the five key candidate genes in renal tissue samples which were generated using “NMF” version 0.24.0 package in R^64^ (https://cran.r-project.org/package=NMF). *CI* confidence interval.

The ROC curve of five key candidate genes for the GSE116626 dataset was presented in Fig. 9A–E. In GSE116626, the AUC values of five key candidate genes were as follows: FOS (AUC: 0.997, 95% CI 0.989–1.000, Fig. 9A), JUN (AUC: 0.890, 95% CI 0.807–0.973, Fig. 9B), EGR1 (AUC: 0.929, 95% CI 0.859–0.998, Fig. 9C), FOSB (AUC: 0.959, 95% CI 0.910–1.000, Fig. 9D), DUSP1 (AUC: 0.937, 95% CI 0.875–0.999, Fig. 9E). The hierarchical clustering for each key candidate gene was shown in Fig. 9F.

**Figure 10:**
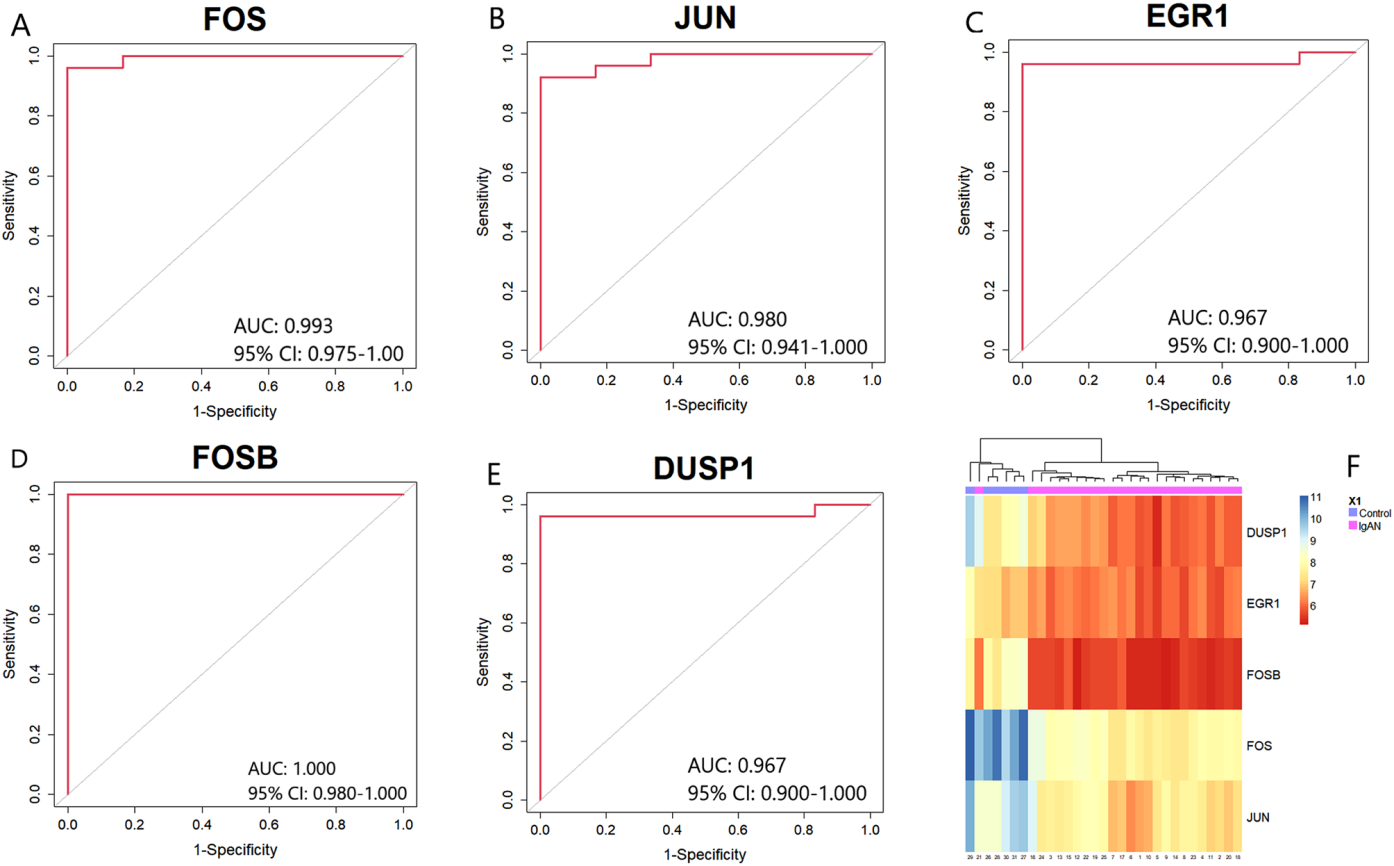
Validation of the five key candidate genes using ROC curves which were generated by pROC package with version 1.18.0 in R^29^ (https://cran.r-project.org/package=pROC) and heatmap for GSE35487 dataset. (**A**) FOS (**B**) JUN (**C**), EGR1 (**D**) FOSB (**E**) DUSP1 (**F**) Heatmap of the five key candidate genes in renal tissue samples which were generated using “NMF” version 0.24.0 package in R^64^ (https://cran.r-project.org/package=NMF).

Similarly, the ROC curve of five key candidate genes for the GSE35487 dataset was presented in Fig. 10A–E. We observed that the AUC values of five key candidate genes were greater than 0.900. The AUC values of these five key candidate genes were as follows: FOS (AUC: 0.993, 95% CI 0.975–1.000, Fig. 10A), JUN (AUC: 0.980, 95% CI 0.941–1.000, Fig. 10B), EGR1 (AUC: 0.967, 95% CI 0.900–1.000, Fig. 10C), FOSB (AUC: 1.000, 95% CI 0.980–1.000, Fig. 10D), DUSP1 (AUC: 0.967, 95% CI 0.900–1.000, Fig. 10E). The hierarchical clustering for each key candidate gene was shown in Fig. 10F. Finally, we recommended that the five key candidate genes (FOS, JUN, EGR1, FOSB, and DUSP1) may be considered as potential genes or key candidate genes for IgAN. Therefore, our findings were validated for both GSE116626, and GSE35487 datasets.

## Discussion

In this study, we evaluated the GSE93798 dataset from GEO database to filter DEGs for IgAN patients and determine the key candidate genes. We identified 348 DEGs (up-regulated: 107 and down-regulated: 241) from GSE93798 that can be easily differentiated IgAN patients from healthy controls (Fig. 2A–B). To validate the pathogenetic process of DEGs, we did gene functional enrichment analysis of DEGs using DAVID. We considered the top five GO terms for BPs, MFs, and CCs, as well as KEGG pathways, that were statistically significantly associated with IgAN patients through DEGs. According to the GO analysis for BP, the DEGs were statistically significantly associated with inflammatory, camp, and cellular responses to lipopolysaccharide, cytokine-mediated signaling pathway, and neutrophil chemotaxis. Among them, some previous studies found response to inflammatory^10,21,30^, response to camp^10,21,22,31^, and cellular response to lipopolysaccharide^10,21^ as highly significant GO terms.

In case of CCs, the top five GO terms were significantly associated with DEGs for IgAN patients, which we got in this study were consistent with previous studies such as extracellular exosome^10,21,30^, extracellular region^10,21,30^, extracellular space^10,21,30^, collagen trimer^21,22^, and blood microparticle^10,21,22^. For MFs, the three MFs supported by previous studies were extracellular matrix structural constituents^22^, transcriptional activator activity, RNA polymerase II transcription regulatory region^10,20–22^, and zinc ion binding^30^. In KEGG pathway analysis, our findings were closely related with previous studies. They showed that glycine, serine and threonine metabolism^10,22^, age-rage signaling pathway in diabetic complications^22,32^, protein digestion and absorption^21,22,30^, IL-17 signaling pathway^22,32^, and osteoclast differentiation^10,21,22,30,32^ were significant pathways for DEGs.

The 348 DEGs were imported to STRING and visualized their PPI network with 206 nodes and 880 edges using Cytoscape. On the basis of degree of connectivity>18, we selected 19 hubs genes from the PPI network, which were showed in Table 3. Two significant modules were selected using MCODE with the cutoff points: MCODE scores $\ge 6$ and number of nodes $\ge 6$. The first module had 10 nodes and 11 nodes were in module 2, which were presented in Table 4 and their PPI network were also presented in Fig. 3B,C. Furthermore, we selected 21 hub module genes by taking the union of module 1 and module 2. To identify the discriminative genes, we applied three ML-based algorithms (SVM, LASSO, and PLS-DA) on 348 DEGs. We selected 35 discriminative genes using SVM (see in Fig. 4), 32 discriminative genes using LASSO (see in Fig. 5C, and 20 discriminative genes using PLS-DA (see in Fig. 6B). We identified five key candidate genes (FOS, JUN, EGR1, FOSB, and DUSP1) from the hub genes, hub module genes, and discriminative genes selected by SVM, LASSO, and PLS-DA (see Fig. 7A) and their PPI network were showed in Fig. 7B. We observed that each key candidate gene could be easily differentiated IgAN patients from healthy controls (Fig. 8A–E). The hierarchical clustering of the key candidate genes revealed that they were able to completely separate IgAN patients from healthy controls (Fig. 8F).

FOS is a component of activator protein 1 (AP-1) transcription factors^33^ that controls the expression of genes involved in cell growth, death, inflammation, and differentiation^30,34,35^. FOS was significantly linked with DNA damage, telomere injury-related aging markers, and neutrophil activation, which also controlled IgAN initiation and evolution^36,37^. A study revealed that FOS was related to the disappearance of podocyte foot processes^38^. Our findings showed that FOS was strongly associated/correlated with IgAN, which was consistent with the previous studies^10,20,22,30,32,36,39^. JUN plays a crucial role in IgAN. It is also an AP-1 transcription factors and one of the most potential factors for IgAN. A study revealed that AP-1 was strongly associated with IgAN^15^. Our study also revealed that JUN was also a potential biomarker for IgAN, which was supported by the previous studies^10,22,30,32,36^.

EGR1 is a zinc-finger protein that plays an important role in cell growth and proliferation. It promotes the epithelial-mesenchymal transition that contributes to diabetic kidney disease^39^. In rhabdomyosarcoma, EGR1 overexpression reduces cell proliferation, motility, and anchorage-independent growth^40^. In our study, EGR1 was one of the top five key biomarkers and significantly associated with IgAN, which was also supported by previous studies^10,20,30^. FOSB is one of the members of the FOS gene family and can be overexpressed in numerous diseases such as IgAN, mesangial proliferation, lupus nephropathy, and so on. Our study reported that FOSB was also a significant biomarker for IgAN. One of the DEGs was DUSP1, a gene linked to fibrosis^20^. DUSP1 is involved in both the human biological response to stress and the negative regulation of cell growth^41^. For hypertensive patients, angiotensin-1-7 increased DUSP1, which reduced fibrosis in resistant arterioles and end-stage organ damage^42^. Our study also reported that DUSP1 was a potential biomarker for IgAN, which was consistent with previous study^43^.

In light of the above mentioned approach, we identified five key candidate genes (FOS, JUN, EGR1, FOSB, and DUSP1) that can easily be differentiated IgAN patients from healthy controls. Therefore, our study suggested that FOS, JUN, EGR1, FOSB, and DUSP1 may function as key biomarkers for the detection and diagnosis of IgAN. These five key candidate genes may play an important role in the development of IgAN and act as potential candidate molecular targets for the diagnosis and treatment of IgAN. This research will be helpful to the readers who will be interested in determining the correlated pathway of IgAN. However, more research into the processes of these genes in IgAN is required.

In the future, we will try to implement our proposed system for the identification of key candidate ncRNA for IgAN and compared our findings with previous studies^44–48^. Furthermore, we will adopt more ML-based and deep learning-based algorithms to identify the potential key candidate genes.

## Methods

### Microarray dataset

In this study, we used three publicly available GEO datasets with accession numbers: GSE93798^49^, GSE116626^50^ and GSE35487^51^, which came from renal biopsies and one can easily be downloaded from the GEO database (www.ncbi.nlm.nih.gov/geo/). The GSE93798 dataset was used to determine the key candidate genes. The GSE93798 dataset was based on GPL22945 platform $[HG-U133\_Plus\_2]$ and included 42 subjects, with 20 IgAN patients and 22 healthy controls. Another two independent datasets: GSE116626 and GSE35487 were used for the validation of key candidate genes. The GSE116626 dataset was based on GPL14951 platform and consisted of 52 IgAN patients and 7 healthy controls. On the other hand, the GSE35487 dataset was based on the GPL96 platform and composed of 25 IgAN patients and 6 healthy controls. Although these datasets were taken from the publicly available GEO repository, being the Human data, all methods were performed in accordance with the relevant guidelines and regulations.

### Identification of DEGs

Using the platform GPL22945, the probe matrix was merged with our gene series matrix by Affymetrix ID and no genes were removed from our database. The DEGs between IgAN patients and healthy controls were identified using the limma package^52^ in R software with version 4.1.2 (https://cran.r-project.org/). The DEGs were selected using the following cutoffs: adjusted probability value (*p*-value) $< 0.05$ and $|\text {logFC}|>1$. Where, FC is the fold change. The DEGs between IgAN and healthy control subjects were analyzed using hierarchical clustering.

### Enrichment analysis of DEGs

The DEGs and top key candidate genes were both selected for GO and KEGG pathway analysis^53^. With these DEGs and top key candidate genes, GO term and KEGG enrichment analysis were obtained using DAVID version 6.8 tools (david.ncifcrf.gov) and a *p*-value $< 0.05$ was chosen as the cut-off criteria.

### PPI network analysis and hub gene identification

We constructed an integrated network among selected DEGs. The STRING version 11.5 online based software (www.string-db.org) was used to make the network^21^. We set a confidence score to $> 0.70$ and a maximum number of interactors to 0 as a cutoff value to build the interaction of DEGs. Then, export the string interaction file and save it in TSV format. We visualized the PPI network on Cystoscape version 3.9.1^54^. To identify the hub genes, we set the degree of connectivity $>18$ as a cutoff value.

### Hub module and its gene identification

MCODE was used to visualize the significant nodes and also partition the network into different modules with degree cut-off $=2$, cluster finding $=$ haircut, node score cut-off $=0.2$, K-score $=2$, and maximum depth $=100$, respectively. To select the most significant modules using MCODE, we set the cutoff values as follows: MCODE scores $\ge 6$ and number of nodes $\ge 6$, respectively. After selecting the significant module, we selected the hub module using the following formula:

1 $$\begin{aligned} \text {Hub Module Genes =}\bigcup _{i=1}^{m}{\text {Genes from Module}}_i \end{aligned}$$

where, m is the number of significant modules. The corresponding genes were considered as hub module genes.

### ML-based discriminative gene selection

After identifying DEGs, we have adopted three supervised ML algorithms as support vector machine (SVM), least absolute shrinkage and selection operator (LASSO), partial least squares discriminant analysis (PLS-DA) to identify the  discriminative genes of IgAN. The brief descriptions of these algorithms are summarized as follows:

#### Support vector machine

SVM^55,56^ is one of the most popular supervised ML algorithms. The aimed of SVM is to determine a hyperplane in a high dimensional space that can easily classified the groups as IgAN patients and healthy controls which needs to solve the following constraint problem:

2 $$\begin{aligned} \max _\alpha \sum _{i=1}^{n}\alpha _i-\frac{1}{2}\sum _{i=1}^{n} \sum _{j=1}^{n}{\alpha _i\alpha _jy_iy_jK(x_i,\ x_j)} \end{aligned}$$

Subject to

3 $$\begin{aligned} \sum _{i=1}^{n}{{y_i}^T\alpha _i}=1, 0\le \alpha _i\le C, i=1,\ldots ,n\ \& \ \forall \ i=1, 2,3,\ldots ,n \end{aligned}$$

The final discriminate function takes the following form:

4 $$\begin{aligned} f(x)= \sum _{i=1}^{n}{\alpha _iK(x_i,\ x_j)}+b \end{aligned}$$

where, b is the bias terms.

In this research, we have used radial basis kernel which is defined as follows:

5 $$\begin{aligned} K(x_i,\ x_j)=\text {exp}(-\gamma \Vert x_i-x_j \Vert ^2) \end{aligned}$$

There were some additional parameters in SVM with RBF kernel, such as cost (C) and gamma $(\gamma )$, called hyper parameters. These hyperparameters were tuned using the grid search method and chose the hyperparameters that provided the highest classification accuracy. In this study, we used SVM as discriminative gene selection algorithm. We will identify the most discriminative genes from a set of DEGs for IgAN patients based on the following steps:

- **Step 1**: Take 80% of the dataset for the training set and 20% of the dataset for the test set.
- **Step 2**: Choose one gene from a list of 348 DEGs.
- **Step 3**: Trained SVM model on the training dataset.
- **Step 4**: Calculate the classification accuracy for this feature.
- **Step 5**: Repeat Step 1 to **Step 4** into five times.
- **Step 6**: Calculate the average of the classification accuracy.
- **Step 7**: Repeat **Step 1** to **Step 6** for all (348) genes.
- **Step 8**: Sort the classification accuracy from the largest to smallest.
- **Step 9**: Select the genes that will produce more than 95.0% classification accuracy.

#### LASSO

LASSO is a supervised learning that is widely used both in biomarker selection and classification problems. We trained a logistic LASSO-based regression model on 348 DEGs to identify the discriminative genes of IgAN using the “*glmnet*” package in R with version 4.1.2^27,57^. To select the optimal parameters, we adopted a 10-fold cross-validation protocol, and the partial likelihood deviance met the minimum criteria. The genes with non-zero coefficients of the LASSO-based logistic regression model are selected as discriminative genes, and we remove the genes with zero coefficients of the LASSO-based model from our analysis.

#### PLS-DA

PLS-DA is one of the most popular supervised ML algorithms. It is widely used not only in dimension reduction algorithms such as PCA, but also in gene selection^58–60^ and classification^61,62^. We utilized PLS-DA while the response variable takes a categorical variable, for example, “1” for yes and “0” for no. It is similar to logistic regression. In this study, we used PLS-DA as a gene selection method to identify the discriminative genes for IgAN patients using the *“mixOmics”* package in R.

### Key candidate genes identification

To identify the key candidate genes and avoid the missing the important genes, we identified the discriminative genes using three ML-based methods (SVM, LASSO, and PLS-DA), the hub genes using the degree of connectivity from PPI network, and hub module genes from significant modules. We identified the key candidate genes using the following formula:

6 $$\begin{aligned} \text {Key Candidate Genes =}\bigcap _{i=1}^{k}{\text {Identification Methods}}_i \end{aligned}$$

where, k is the number of potential gene identification methods.

## Data Availability

The datasets generated and/or analysed during the current study are available in the Gene Expression Omnibus (GEO) repository with accession numbers: GSE93798, GSE116626 and GSE35487. Using these accession numbers, one can easily download these datasets from the following link: www.ncbi.nlm.nih.gov/geo/.
